# Prognostic Signatures and Therapeutic Value Based on the Notch Pathway in Renal Clear Cell Carcinoma

**DOI:** 10.1155/2022/1669664

**Published:** 2022-01-20

**Authors:** Ziyao Li, Shiyong Xin, Shuanbao Yu, Jing Liang, Xuepei Zhang

**Affiliations:** ^1^Department of Urology, The First Affiliated Hospital of Zhengzhou University, Zhengzhou, Henan 450052, China; ^2^School of Electrical Engineering of Zhengzhou University, Zhengzhou, Henan 450052, China; ^3^Department of Urology of Shanghai Tenth People's Hospital, School of Medicine, Tongji University, Shanghai 200092, China; ^4^Department of Urology, The First Affiliated Hospital, and College of Clinical Medicine of Henan University of Science and Technology, Luoyang 471003, China; ^5^Key Laboratory of Precision Diagnosis and Treatment for Chronic Kidney Disease in Henan Province, Zhengzhou 450052, China

## Abstract

The Notch family of genes encodes a group of highly conserved cell surface membrane receptors, which are involved in one of the key pathways that determine cell growth, differentiation, and apoptosis in embryonic tissues. Furthermore, abnormal expression of Notch genes is closely related to the occurrence and development of several cancers. To date, no specific treatment of RCC has been reported to relate to the Notch pathway. Therefore, we detected Notch pathway genes in series of tumors, as well as potential compounds targeting the Notch pathway, with a focus on the mechanism of Notch pathway action in kidney renal clear cell carcinoma (KIRC). Samples from KIRC patients were divided into three clusters based on the mRNA expression of Notch pathway genes. In addition, we investigated the potential targets of the Notch pathway, predicted the IC_50_ of several classical targeted therapies, and analyzed their correlation with the Notch pathway. Finally, LASSO regression analysis was performed to build a model to predict survival in KIRC patients. These results suggest that therapies targeting the Notch pathway could be more efficiently studied based on the Notch score and that we can predict the prognosis of patients with KIRC based on the expression of Notch pathway genes. Most importantly, these results may provide a solid theoretical basis for future research on therapeutic targets for patients with KIRC.

## 1. Introduction

The Notch signaling pathway plays an important role in cancer biology and is the focus of research on targeted therapy for cancer. The Notch pathway was first observed in Drosophila in 1914, and related genes were discovered in 1986 [1]. The Notch signaling pathway is mainly composed of four parts, i.e., receptors, ligands, CSLDNA-binding protein, and downstream genes. The Notch signaling pathway is different from other important pathways, such as Wnt and TGF-*β*, and comprises receptors and ligands that mediate the activation of two cells after contact [2]. In humans, the Notch receptors (Notch 1, 2, 3, and 4) interact with the Notch ligands (Jagged 1, 2; Delta 1, 3, 4, the difference between these two types of ligands is the presence of a cysteine-rich region [3], in addition to the differences in function [4]). The Notch receptor is hydrolyzed by ADAM-*γ*-secrete to produce NICD, which binds to CSLDNA-binding proteins and activates downstream target genes [5]. The Notch signaling pathway has been shown to play an important role in homeostasis during cell development and the development and progression of diseases, especially those of cancer, with varying roles in various cancer tissues. For example, in small cell lung cancer, bladder cancer, and other cancers, the Notch pathway intermediaries, Notch 1, 2, 3, and 4, Mastermind-like (MAML), NICASTRIN, and other genes can serve as protooncogenes, leading to the hyperactivation of the Notch pathway. Notch 1, 2, 3, FBXW7, and other genes serve as tumor suppressor genes in T-ALL and breast cancer [6]. Kidney cancer is one of the cancers whose incidence has been on the rise in recent years. It was estimated that the global incidence of this cancer would exceed 70,000 cases by 2020 [7]. Renal cell carcinoma (RCC) is the main type of kidney cancer, while clear cell renal cell carcinoma (ccRCC) cases comprise a small percentage of RCC cases (75-80%) [8, 9]. Advances and innovations in the treatment of ccRCC can benefit the majority of patients with renal cancer. In recent years, the use of targeted therapy for ccRCC has become a mainstream trend in clinical treatment, including therapies that target the mTOR pathway [10], ferroptosis [11], and lipid metabolism [12], and other strategies that targeting other pathways or processes. Therefore, we considered whether targeting the Notch signaling pathway could provide more possibilities for the treatment of ccRCC.

In this study, we investigated the role of the Notch signaling pathway in ccRCC and related therapeutic targets through bioinformatic analysis and established a Notch-related prognosis model of ccRCC by using a LASSO estimate for linear regression to screen related genes, thereby providing a relatively meaningful strategy for the treatment of ccRCC. We used gene set enrichment analysis (GSEA) to screen out more than 40 genes involved in the Notch pathway and were differentially expressed in ccRCC. Using these genes, we classified the samples obtained from The Cancer Genome Atlas (TCGA) into three clusters, and we continued our research based on these three clusters. We identified potential therapeutic compounds and carried out classic anticancer drug effect analysis, histone modifications, and immune infiltration analysis. Finally, using LASSO regression analysis, we selected 14 genes from more than 40 genes to build a prognosis model, reducing the influence of the selected genes (multicollinearity). We further used the e-MTAB-1980 dataset from the ArrayExpress database for model verification. Moreover, we verified the differential expression of related genes encoding proteins in ccRCC in the model based on the relevant immunohistochemical results. All data acquisition, collation, and analysis, including statistical analysis and tests, were carried out using R. While introducing the experimental ideas, we will also indicate the specific R software package used.

## 2. Materials and Methods

### 2.1. Data Acquisition and Analysis Based on TCGA

The RNA-SEQ and clinical data of ccRCC were obtained from TCGA (https://portal.gdc.cancer.gov/), including 72 normal tissue samples and 539 ccRCC tissue samples. All Notch pathway genes were identified using Wiki Pathways from GSEA. Ultimately, 47 Notch pathway genes related with 32 types of cancer were identified. We also obtained the data for CNV, SNV, and Notch gene expression from TCGA (https://portal.gdc.cancer.gov) [13–18]. Perl was used for analyzing Notch gene-related data, and TBtools were used for data visualization.

### 2.2. Correlation Analysis of Drugs or Compounds with Notch Pathway Genes

To identify which drugs or compounds are useful for Notch-related tumor therapy, we performed correlation analysis using Connectivity Map Build02 (CMap)^14^, a resource that uses cellular responses to perturbation to find relationships between diseases, genes, and therapeutics. CMap (Build 02) contained the expression profile of 6,100 genes from five human cell lines treated with 1309 different doses of drug, and a ranked list of compounds with connectivity scores between -1 and 1 was obtained by comparing disease characteristics with all reference expression profiles of the chemicals. There was a high degree of negative correlation between the disease characteristics and the expression profile associated with the compound, suggesting that the compound may have therapeutic effects. The CMap database has successfully led to drug repurposing for a variety of diseases, including lung cancer, breast cancer, muscular dystrophy, acute myeloid leukemia, Parkinson's disease, and Alzheimer's disease [15, 16]. In this study, bioinformatics and CMap analysis were used to screen out the key pathogenic genes and candidate small molecule therapeutic against the Notch pathway in cancer, providing new ideas for the treatment of KIRC. Ultimately, 14 differential characteristics and expression of mRNA were obtained for further research through differential expression analysis of Notch pathway genes. *p* < 0.05: statistical significance.

### 2.3. Analysis of Correlation of the Gene Enrichment Scores and Gene Clusters

To further display the differences in gene expression among the samples, we constructed a Notch-score model to classify mRNA expression in tumor tissues into three groups, i.e., high expression of the Notch pathway genes (cluster 1), normal expression of the Notch pathway genes (cluster 2), and low expression of the Notch pathway genes (cluster 3), according to the mRNA expression in the samples. In addition, we used a violin diagram to describe the relationship between gene enrichment scores and the expression levels of the three clusters. In RStudio, we used the “gplot” package for gene cluster analysis. Meanwhile, the survival differences of the three clusters were analyzed, and their survival curves were analyzed using the “survival” package in RStudio. Furthermore, we investigated the relationship between Notch genes and the clinicopathological features of patients with KIRC. *p* < 0.05: statistical significance.

### 2.4. Analysis of Notch Pathway Gene Expression and Tumor Drug Therapy Based on Genomics of Drug Sensitivity in Cancer

To clarify the relationship between Notch pathway gene expression and tumor drug therapy, the Genomics of Drug Sensitivity in Cancer database (GDSC; https://www.cancerRxgene.org) was used to predict chemotherapy response. As one of the biggest public resources for information regarding cancer drug sensitivity, drug reactions, and molecular targets, GDSC enables the identification of potential therapeutic targets to improve the treatment of cancer. GDSC currently hosts nearly 75,000 experimental data on drug sensitivity, documenting the responses of nearly 700 cancer cell lines to 251 anticancer drugs. Focusing on identifying molecular targets for drug sensitivity through a web portal, GDSC databases can be query-based to obtain graphical representations of specific anticancer drugs or cancer gene data. Furthermore, the GDSC database integrates a large set of drug sensitivity and genomic data. In this study, several therapeutic agents targeting KIRC were collected, and the “Prophet” package in R was used to conduct the prediction process. Meanwhile, the ridge regression method was used to estimate the semimaximum inhibitory concentration (IC_50_) of the samples [17, 18]. A smaller IC_50_ always means a lower semi-inhibitory mass concentration of the drug in cancer cells, which indicates that the cancer cells are more sensitive to the drug. Furthermore, 10-fold crossvalidation was performed to estimate the accuracy. *p* < 0.05:statistical significance.

### 2.5. Effect of Differentially Expressed Oncogenes in the Notch Pathway

The Notch signaling pathway plays an important role in regulating tumor cell proliferation, differentiation, and apoptosis. Abnormal activation of the Notch signaling pathway can promote the occurrence and development of several cancers. Chromatin modifications, such as histone methylation, acetylation, and ubiquitination, are key epigenetic mechanisms that regulate gene transcription. Some classical oncogenes and histone modification-related genes in the Notch pathway may influence the regulation of the Notch pathway. In our study, the expression levels of different oncogenes in the three clusters of the Notch pathway were examined and presented as heat maps. *p* < 0.05: statistical significance.

### 2.6. Immune Cell Infiltration and Immunotherapy

To provide a theoretical basis for the clinical application of immune markers and immunotherapy in ccRCC, we investigated the immune response genes in tumor tissues and adjacent tissues and immune cell infiltration in tumor tissues. The correlation of immune cell infiltration in ccRCC samples from TCGA was analyzed using single-sample gene set enrichment analysis (ssGSEA) [19, 20]. Immune gene set analysis was performed, and immune scores were evaluated. The ssGSEA algorithm was used to calculate the degree of immune infiltration in each sample and to study the gene signals expressed in 29 types of immune cells and regulatory cells related to innate and adaptive immunity. After downloading the GMS-format geneset data required for analysis, the R package GSVA was used to score the samples for immunity, including 29 immune-related scores. Finally, we obtained four classic immunomodulators, DCs, mast cells, Tfh, and T1IR, and used the “GGSCATterStats” package to plot scatter plots showing their specific correlation with the Notch score. *p* < 0.05: statistical significance.

### 2.7. Analysis of the Prognosis of KIRC Based on LASSO Regression

To determine the expression level of the Notch pathway gene in normal and KIRC tissues, a heat map was used. In addition, “corrplot” was used to describe the coexpression relationship between any two Notch pathway genes in KIRC. Univariate Cox regression was used to analyze the genes associated with prognosis, and a prognostic model was constructed using LASSO regression and multivariate Cox regression. Univariate and multivariate Cox analysis verified that the Notch gene prognostic correlation model was an independent prognostic factor, and receiver-operating curve (ROC) analysis further verified its accuracy with respect to predicting survival. A risk score was calculated for each sample based on the expression level of relevant genes in the prognostic model, and all samples were divided into high-risk and low-risk groups by the median risk score. The optimal cut-off value was selected using the R software “SurvMiner” package, and the “Survival” package in RStudio was used to compare the overall survival difference between high-risk and low-risk groups. *p* < 0.05: statistical significance.

In the HR analysis, univariate and multivariate Cox regression models were used to analyze the relationship between clinicopathologic features and risk score. To show the multiple attributes of the statistically significant protective and risk genes, we used a Sankey diagram drawn with the “ggalluvial” package. We used RStudio for all statistical analyses. Statistical significance was set at *p* < 0.05.

### 2.8. Model Verification Based on ArrayExpress Database and Relevant Immunohistochemical Results

To verify the model, the e-MTAB-1980 dataset in the ArrayExpress database (https://www.ebi.ac.uk/arrayexpress/experiments/E-MTAB-1980/) comprising 101 case samples was used to verify the value of the model in predicting the prognosis of KIRC. The relevant online atlas of proteins (https://www.proteinatlas.org/) provided by Sjöstedt et al. [21] and Uhlénet al. [22] was used to verify whether or not the expression of model's proteins was consistent with the expression of the corresponding model's genes.

## 3. Results

### 3.1. CNV and SNV of the Notch Pathway Genes in Cancers according to TCGA

**Figure 1:**
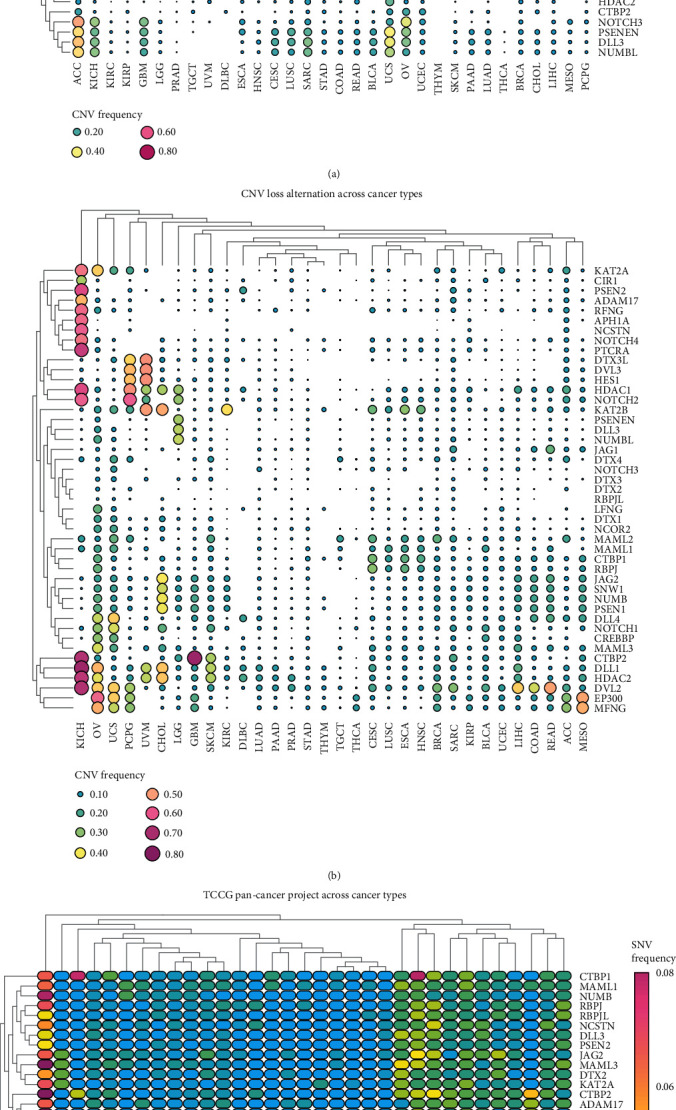
(a, b) CNV frequencies of 47 Notch pathway genes in 32 tumor types from TCGA. The color bar on the right represents gain or copy number, with pink representing high copy frequency and green representing low copy frequency. (c) SNV frequencies of 47 Notch pathway genes. The color bar on the right shows the degree of SNV, with pink representing high frequency and blue representing low frequency.

A total of 47 Notch pathway genes were detected from 32 types of malignant tumors in TCGA, and CNV and SNP of these genes were analyzed by R. We found that among the 32 types of malignant tumors, most tumors showed no CNV gain or loss (Figures 1(a) and 1(b)), and most CNVs fluctuated around 0.2. Notch gene CNV gain frequency > 0.04 and corresponding tumors were as follows: ACC: MAML1, DTX3, DTX1, and NCDR2; KIRP: DTX2, LFNG, and KAT2A; GBM: DTX2 and LFNG; UVM: NOTCH4 and PTCRA; HNSC, CESC, and LUSU: DVL3 and HES1; COAD: RBPJL; READ: LENG and RBPJL; UCS: APH1A, PSENZ, RFNG, and RBPJL; OV: DVL3, HES1; BRCA, CHOL, and LIHC: PSEN2, APHIA, and NCSTN; Notch genes with CNV loss frequency > 0.05 and corresponding tumors were as follows: KICH: KAT2A, PSEN2, RFNG, APHIA, NLSTN, NOTCH4, PTCRA, HDAC1, NOTCH2, CTBP2, DLL1, HDAC2, and DLL2; OV: EP300; PCPG: NOTCH2; GBM: CTBP2. In addition, we found that most of the Notch pathway genes had SNV in UCEC, and the SNV frequency was almost greater than 0.04. Part of the Notch pathway genes in DLBC, SKCM, COAD, STAD, BLCA, CESC, ESCA, HNSC, LUAD, and READ had SNV, and SNV frequency was greater than 0.04. The SNV frequency of the Notch pathway genes in tumors was less than 0.02 (Figure 1(c)). We further observed mutations in the Notch pathway gene in KIRC and found that CNV and SNV of the Notch gene in KIRC were significantly low, fluctuating between 0 and 0.02.

### 3.2. The Role of Different Notch Genes in Different Cancers and the Mechanisms of Action of Each Compound Targeting the Notch Pathway

**Figure 2:**
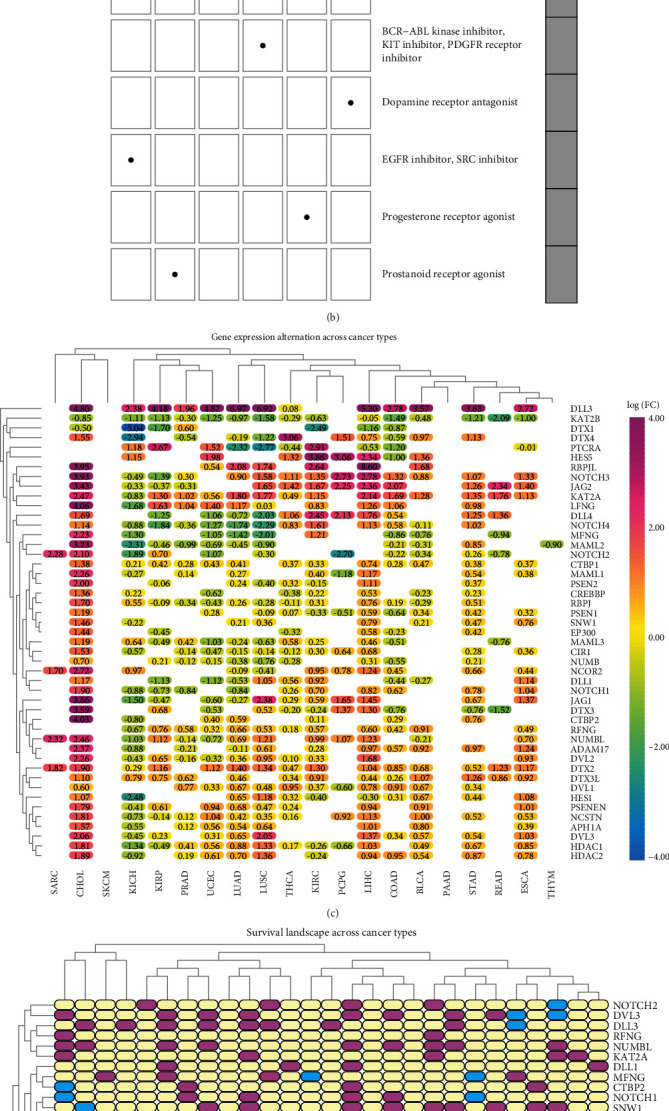
(a) Heatmap shows the enrichment score for each compound for each type of cancer according to the CMap. The color bar represents different enrichment scores: Blue: positive and red: negative. (b) Heatmap shows the mechanisms (column) shared by each compound (row) according to the CMap. (c) The changes in the expression of 47 Notch pathway genes among 32 different types of cancer. The color code bar shows the corresponding value of log2 (FC) on the right side. (d) The role of different genes in different cancers. Pink indicates risk genes, blue indicates protective genes, and white indicates no statistical significance.

A Connectivity Map (CMap) [23] was used to study the relationship between genes and compounds to determine compounds that may target or regulate Notch pathway genes. We identified 20 related compounds that acted on 16 types of malignant tumors using the compound enrichment score (Figure 2(a)). Further, we used the CMap mode-of-action (MoA) to analyze the mechanism of action of the six compounds, and found that each of the six compounds had an independent mechanism of action (Figure 2(b)): galantamine is an acetylcholinesterase inhibitor, and imatinib can inhibit BCR-ABL kinase, KIT, and DGFR receptors. Triflupromazine, a dopamine receptor antagonist, inhibits both EGFR and SRC. Norprogesterone is known as a progesterone receptor agonist, and Denoprost excites prostaglandin receptors. The Notch pathway plays an important role in tumor occurrence and development. Studies have shown that the Notch pathway can inhibit the transcription of tumor cells or promote the effects of tumor cells, i.e., it can act as a cancer suppressor or promoter [6]. In our study, we further divided the Notch pathway genes into protective genes and risky genes using the information in TCGA and investigated the relationship between Notch pathway gene expression and patient survival (genes with high expression in tumor and prolonged patient survival as protective genes, contrary to risky genes), determining their role in the tumor; we found that the expression of 47 Notch genes altered in 32 malignant tumors (Figure 2(c)). The expression of most genes of the Notch pathway in KIRC was significantly higher than that in normal kidney tissue. Further analysis revealed that most genes play a protective role in kidney cancer. We found an interesting phenomenon in which both protective and risky genes were 12 (Figure 2(d)), which is contrary to the report in an article that Notch is a cancer pathway. This strange phenomenon aroused our interest, so we conducted a further biometric analysis to understand the mechanism.

### 3.3. Correlation between the Notch-Score and the Clinicopathological Characteristics of KIRC

**Figure 3:**
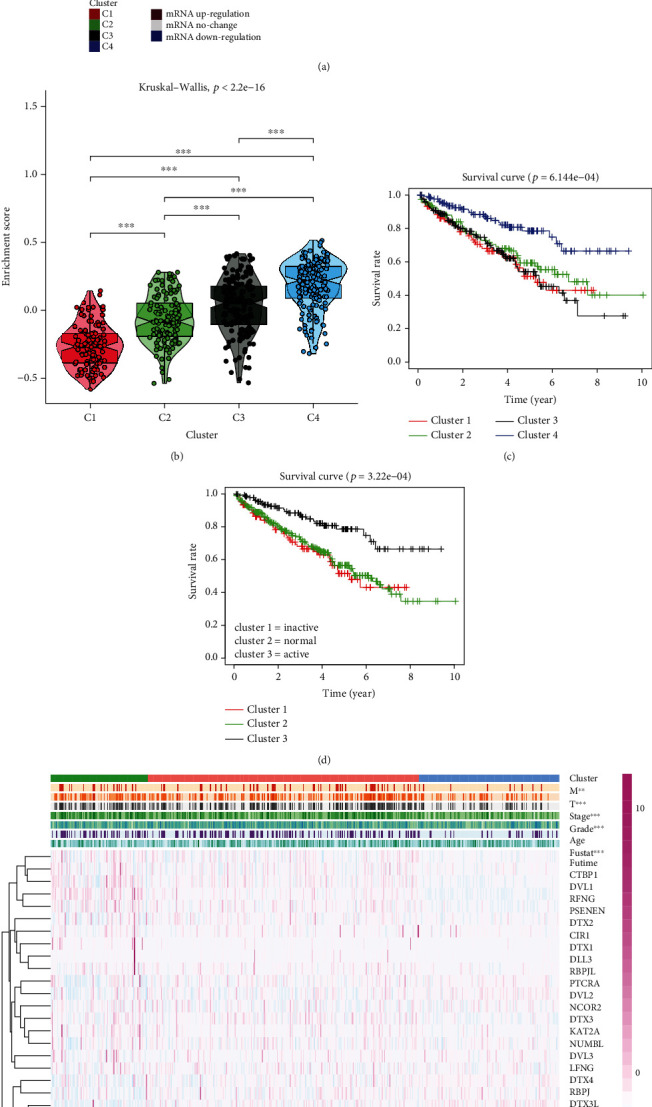
(a) Notch pathway genes were divided into 4 clusters. According to the Notch-score, cluster 1 (red), cluster 2 (black), cluster 3 (green), and cluster 4 (Blue) were ranked from lowest to highest. (b) Violin plot shows the enrichment score of 4 clusters. (c, d) Survival curves of four clusters. Since the distinction is not clear, we merge clusters 2 and 3. The survival curve of the three clusters is shown in the plot. (e) Heatmap of the correlation between the Notch-score and the clinicopathological characteristics (^∗^*p* < 0.05, ^∗∗^*p* < 0.01, ^∗∗∗^*p* < 0.001).

To further study the relationship between Notch pathway genes and KIRC, according to the mRNA expression level of Notch pathway genes obtained from TCGA, we divided the 47 Notch genes in the KIRC samples into four groups (following the Notch score from low to high) as follows: cluster 1, cluster 2, cluster 3, and cluster 4 (Figure 3(a)). We demonstrated the differences in the Notch score among the four clusters using a violin diagram (Figure 3(b)). The Notch score was significantly different (*p* < 0.05) among the four clusters. Furthermore, we described the survival curves of the four clusters (Figures 3(c) and 3(d)) and combined cluster 2 and cluster 3 into a new cluster 2. Through the analysis of the survival curve, it was found that there were significant differences in the survival rates of KIRC patients in the three new clusters. The prognosis of cluster 3 was significantly better than that of cluster 1 and cluster 2, which is similar to that depicted in Figure 2(c). Most Notch genes are highly expressed in KIRC, and all the patients with high Notch scores had longer survival. These data suggest that Notch may play a role as a tumor suppressor gene in KIRC. Finally, using information from TCGA, we further analyzed the relationship between Notch scores and the clinicopathological features of KIRC (Figure 3(e)). We found that the Notch scores were significantly related to the tumor, stage, metastasis, and fustat of KIRC, and a higher Notch score was associated with lower tumor grade, stage, and prognosis. There was a statistically significant difference (*p* < 0.05); the Notch score was not associated with age or Fiume (*p* > 0.05). This further suggests a protective role of the Notch pathway in KIRC.

### 3.4. Drug Sensitivity Analysis of Notch Pathway Genes in KIRC Based on GDSC Data

**Figure 4:**
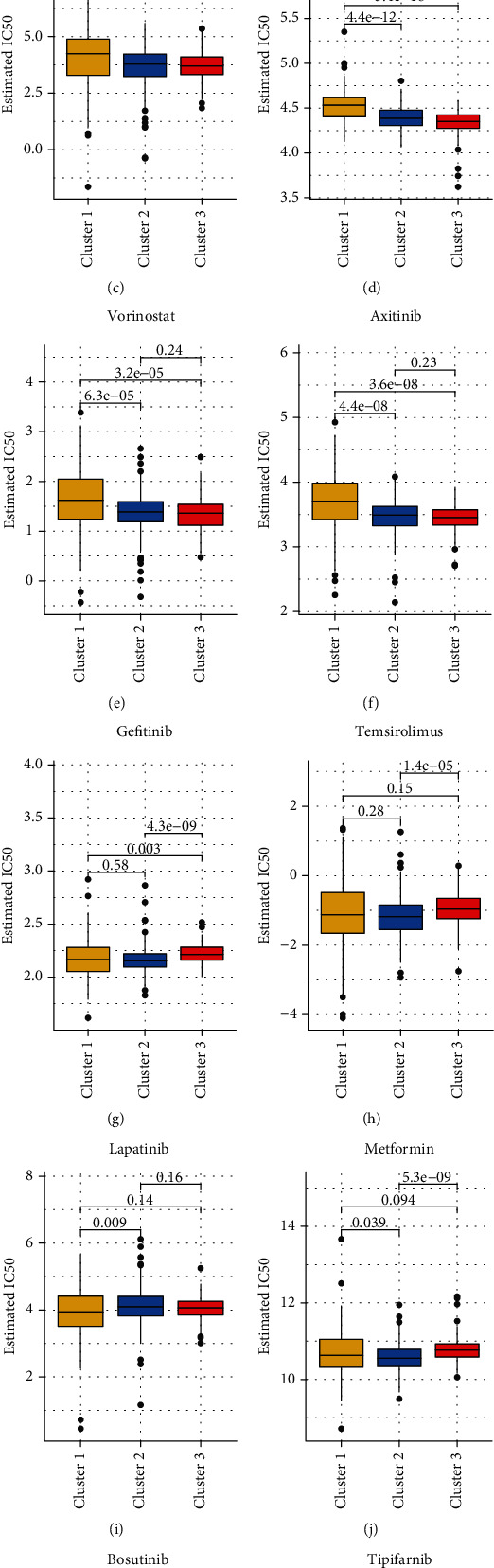
The estimated IC50 for 12 types of common chemotherapeutic agents are shown in the plot for cluster 1, cluster 2, and cluster 3. The 12 types of chemotherapeutic agents are pazopanib, sorafenib, sunitinib, nilotinib, vorinostat, axitinib, gefitinib, temsirolimus, lapatinib, metformin, bosutinib, and tipifarnib.

At present, the treatment of advanced renal cancer mainly relies on molecular targeted drugs and novel immune checkpoint inhibitors. Molecular targeted therapy of cancer is based on the molecular biology of cancer, taking tumor-related molecules as targets, using specific agents or drugs for target molecules for treatment. Since 2006, 11 types of targeted drugs, sorafenib, sunitinib, bevacizumab+IFN, ticsirolimus, everollimus, acitinib, pazopanib, capotinib, navumab, lenvatinib, and erlotinib, have been recommended by the NCCN to use as a first- or second-line treatment for metastatic kidney cancer. According to their targets, these 11 targeted therapy drugs are classified into VEGF inhibitors, i.e., sorafenib, sunitinib, bevacizumab, acitinib, prazopani, capotinib, and lovaritinib; MTOR inhibitors, i.e., tesirolimus, everolimus; Pd-1 inhibitor, Navumab, and EGFR inhibitors, i.e., erlotinib [24–27]. As mentioned above, the Notch pathway plays an important role in the occurrence and development of tumors. Our data also show that the Notch pathway genes might play a protective role in KIRC. Is there any relationship between the Notch pathway gene and the current targeted drugs that can effectively treat advanced renal cancer? To clarify this question, we further studied the relationship between the Notch gene and the IC_50_ of 12 commonly used targeted drugs. A ridge regression model was built to predict the IC_50_ of the drugs against tumor using the GDSC database. The Notch gene is associated with most targeted therapies as shown in Figure 4, the sensitivity of each Notch gene cluster toward each drug is as follows: for pazopanibm, cluster 3 is better than cluster 2; for sorafenib, cluster 1 is better than cluster 2, and cluster 2 is better than cluster 3; for sunitinib, cluster 3 is better than cluster 1, and cluster 2 is better than cluster 1; for nilotinib, cluster 3 is better than cluster 2, and cluster 2 is better than cluster 1; for vorinostat, cluster 3 is better than cluster 1, and cluster 2 is better than cluster 1; for axitinib, cluster 3 is better than cluster 1, and cluster 2 is better than cluster 1; for gefifitinib, cluster 2 is better than cluster 3, and cluster 1 is better than cluster 3; for temsirolimus, cluster 2 is better than cluster 3, and cluster 1 is better than cluster 3; for lapatinib, cluster 1 is better than cluster 2; for metformin, cluster 2 is better than cluster 1, and cluster 2 is better than cluster 3; for bosutinib, cluster 2 is better than cluster 3, and cluster 3 is better than cluster 1; for tipipifarnib, cluster 1 is better than cluster 3, and cluster 2 is better than cluster 3. Through our study, the correlation between the therapeutic effects of commonly used targeted drugs and Notch genes might be well understood, which may be helpful for advanced KIRC treatment in the future.

### 3.5. Correlation of Notch Pathway Genes with Potentially Targetable Classical Genes, Sirtuin Family Genes, and HDAC Family Genes

**Figure 5:**
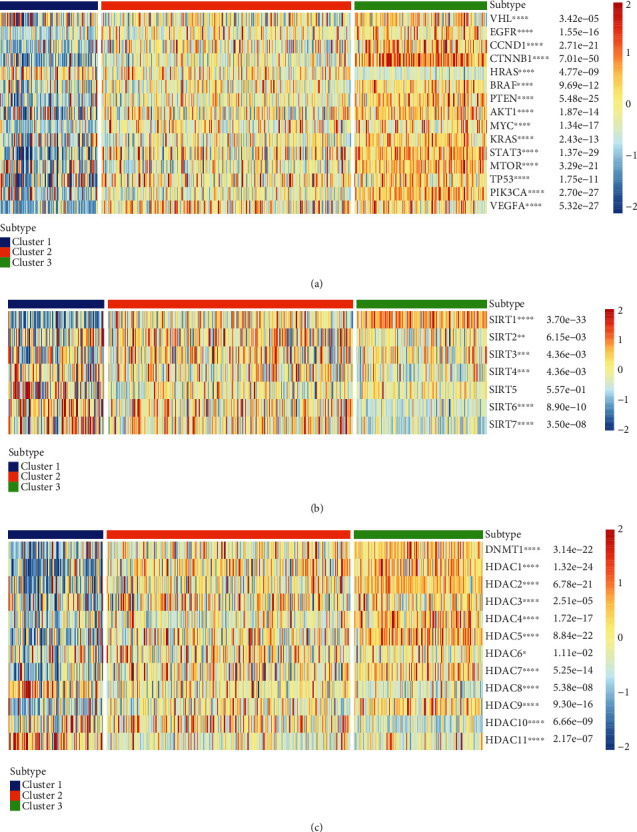
(a) Correlation between Notch genes and potentially targetable classical genes. (b) Correlation between Notch genes and Sirtuin family genes. (c) Correlation between Notch genes and HDAC family genes.

Histone acetylation and deacetylation play important roles in regulating gene expression. In addition to the classical histone deacetylation enzymes (HDACs) of classes I and II, there is also a special class of HDACs (class III HDAC, Sirtuin). According to the characteristics of the substrates, it is speculated that the physiological function of human Sirtuin protein may be involved in the regulation of the balance of cell survival and death under stress conditions. In contrast, metabolism regulation affects the development, differentiation, aging, and other physiological processes and is closely related to cancer [26]. SIRT5-mediated SDHA desuccinylation promotes clear cell RCC tumorigenesis [28]. In addition, the SIRT family shows a differentially expressed organization in RCC [29]. Deacetylation of the tail area of histones could cause DNA to bind more tightly to the histone core area, preventing the promoter region from being activated and ultimately inhibiting transcription [30, 31]. In our study, we further clarified the relationship between the Notch pathway gene and common oncogenes in KIRC, and the correlation of the Notch pathway genes with the Sirtuin family and HDAC family proteins. The results showed that AKT1, MYC, and VEGFA was highly expressed in cluster 3 (the exception being HRAS), but they were low expression in cluster 1 in KIRC, suggesting the role of these molecules as oncogenes. However, common tumor suppressor genes, such as VHL and PTEN, were highly expressed in cluster 3 with low expression in cluster 1, much like the oncogenes (Figure 5(a)). In addition, the results of the relationship between the Notch pathway genes and Sirtuin family genes show that SIRT1 was highly expressed in cluster 3 but had low expression in cluster 1. SIRT2 and SIRT3 were highly expressed in cluster 2, but had low expression in clusters 1 and 3 with no significant difference. SIRT4, SIRT5, SIRT6, and SIRT7 were all highly expressed in cluster 3 but had low expression in cluster 1. This suggests that the Sirtuin family plays different roles in the KIRC (Figure 5(b)). Furthermore, the results of the relationship between the Notch pathway genes and HDAC family genes show that DNMT1, HDAC1–HDAC7, and HDAC9 were all highly expressed in cluster 3 but had low expression in cluster 1. HDAC8 and HDAC11 were highly expressed in cluster 1 but with low expression in cluster 3. HDAC10 had the highest expression in cluster 2 and higher expression in cluster 1 than in cluster 3. Similar to the Sirtuin family, it is suggested that the HDAC family also plays different roles in KIRC (Figure 5(c)).

### 3.6. Analysis of the Correlation between the Notch Pathway Genes and Immune Infiltration

**Figure 6:**
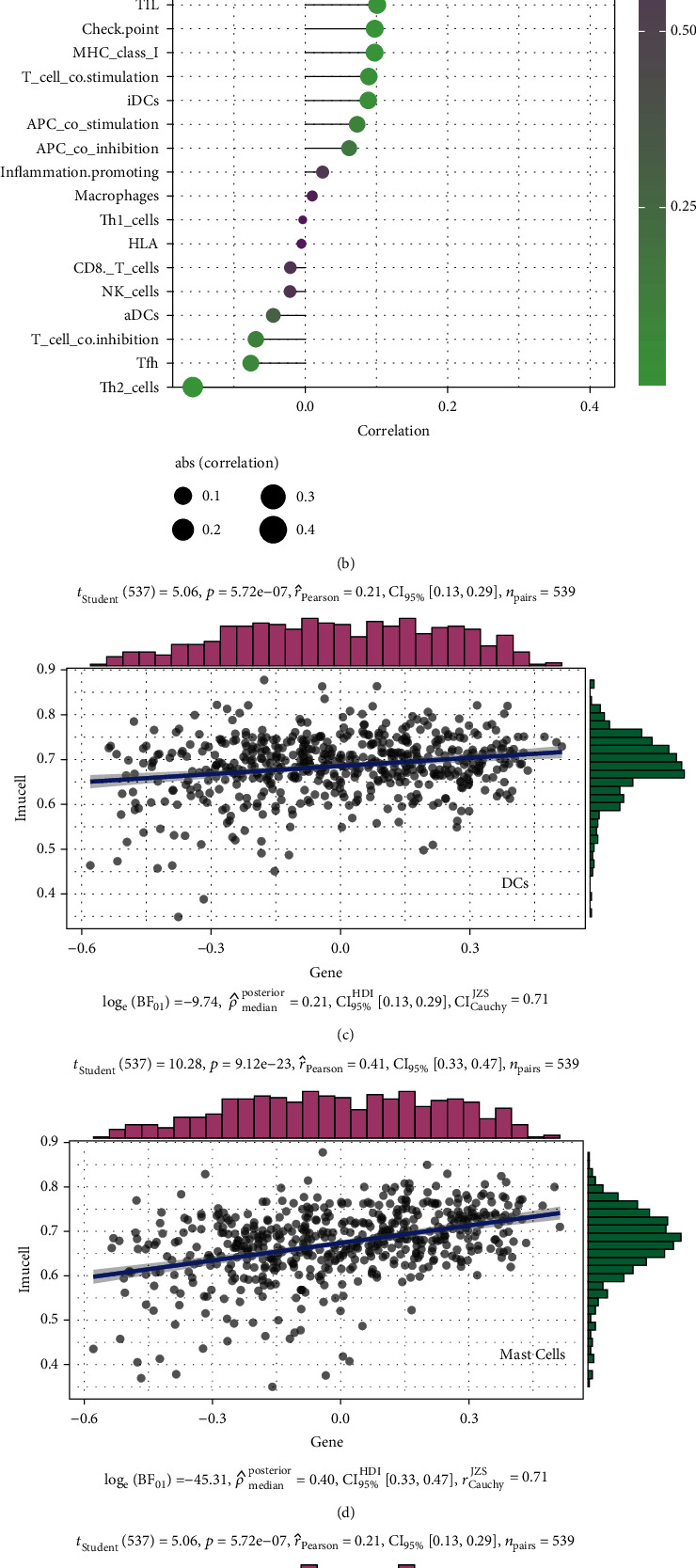
(a) Heatmap showing the correlation between Notch pathway genes and immune-infiltrating agents. Pink represents positive, and blue represents negative (^∗^*p* < 0.05, ^∗∗^*p* < 0.01). (b) In the plot depicting the degree of correlation, the area of the sphere represents the abs (correlation) and the color represents the *p* value. (c–f) The scatter plot depicting the specific relationship between four immune infiltration-related agents and the Notch-score, and it can be seen from the figure that they are all positively correlated.

The tumor microenvironment (TME) is a two-way interaction between tumor cells and stromal cells, dynamic with the role of complex networks. During the development of tumor cells, immune escape occurs and further develops into metastases, and the TME provides the necessary cellular and molecular environment for this dynamic process. Different degrees of immune cell infiltration exist in the tumor microenvironment of renal cancer, and the immune cell infiltration pattern is closely related to the survival and clinical stage progression of renal cancer. In the future, better targeted drugs can be developed according to the immune cell infiltration pattern [32–34]. In this study, we further explored the relationship between 47 Notch pathway genes and 29 immune infiltration-related factors and cells. The results show that PTCRA, MFNG, LFNG, Notch1-3, DTX3L, DTX2, CIR1, APH1A, and ADAM17 are positively correlated with most immune-infiltrating agents. In contrast, SNW1 RFNG, NUMB, Notch4, MAML3, KAT2A, DVL2, DVL1, DLL1, and CTBP2 are negatively correlated with immune infiltration (Figure 6(a)). Most immune-infiltrating components were positively correlated with the Notch pathway genes, including the type-II-IFN response, mast cells, DCs, and CCR. A few, including DCs, Tfh, Th2-cells, and T-cell-coinhibition showed a negative correlation with the Notch pathway genes (Figure 6(b)). Finally, we analyzed the correlation between four immune-infiltrating components (DCs, mast cells, T-helper cells, and type-I-IFN response) and Notch scores. The results show that the responses of DCs, mast cells, T-helper cells, and type-I-IFN are positively correlated with the Notch score (Figures 6(c)–6(f)).

### 3.7. KIRC Patient Prognosis Analyzed through LASSO Regression

**Figure 7:**
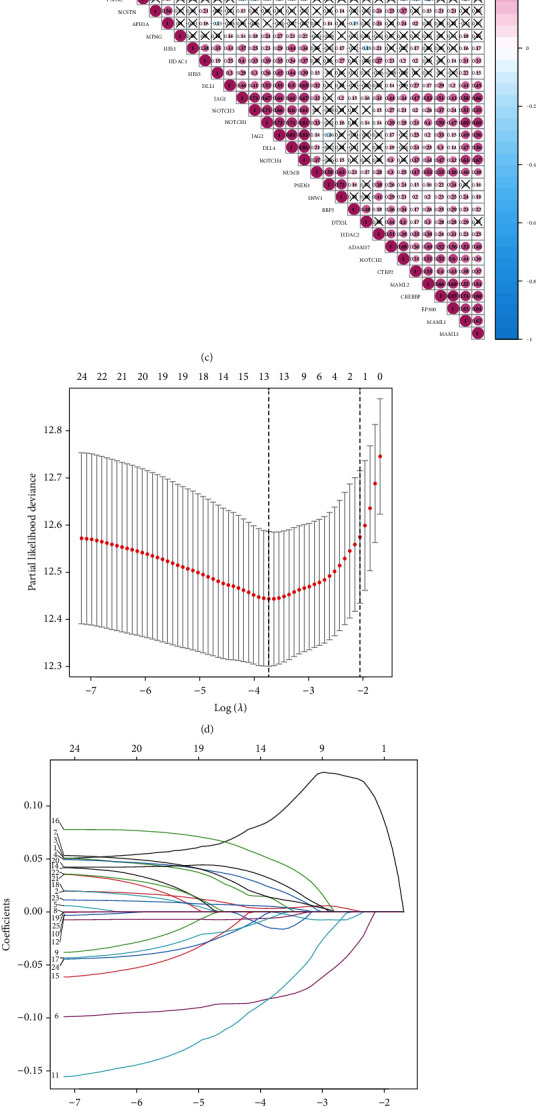
(a) Expression of 47 Notch pathway genes in KIRC patients. In the color bar on the right side, pink represents upregulation, and blue represents downregulation. N (blue) is the normal sample; T (red) is the tumor sample (^∗^*p* < 0.05, ^∗∗^*p* < 0.01, ^∗∗∗^*p* < 0.001). (b) Plot showing hazard ratio (HR) analysis with 95% confidence interval (CI) and *p* values. (c) Plot showing the results of the coexpression analysis of 47 Notch pathway genes. (d) LASSO coefficient profiles of Notch pathway genes in KIRC. (e) 14 genes were selected by LASSO Cox regression analysis. (f) Correlation between 14 selected genes and the clinicopathological characteristics in the two groups. The color bar shows the expression of genes, pink represents upregulation, and blue represents downregulation (^∗^*p* < 0.05, ^∗∗^*p* < 0.01, ^∗∗∗^*p* < 0.001). (g) Survival curve obtained based on this model. Pink and blue correspond, respectively, to the high-risk group and the low-risk group. (h–k) ROC curve of 3, 5, 7, and 10 years.

Notch plays a different role in different tissues and cells and may promote or inhibit cancer depending on the tumor type and other signaling pathways. However, Notch has a cancer-promoting role in most tumors. Studies have suggested that Notch expression level is associated with the prognosis of KIRC, and high Notch expression might indicate poor prognosis [6, 7]. In our study, we compared the differences in the expression of 47 Notch pathway genes in normal kidney tissues and renal cancer tissues. The results show that the expressions of 36 Notch pathway genes is abnormal in 72 normal kidney tissues and 539 KIRC samples obtained from TCGA (Figure 7(a)). Further hazard ratio (HR) analysis revealed that 25 Notch pathway genes were associated with KIRC progression, 14 of which were significant, including KAT2B, KUMB, NUMBL, DVL3, and JAG1 (Figure 7(b)). Results of gene coexpression analysis indicate that there is a coexpression relationship between Notch pathway genes (Figure 7(c)). We further used LASSO regression to establish a predictive model to analyze the value of Notch pathway genes in predicting the prognosis of patients with KIRC. Therefore, we selected 14 genes (HDAC1, JAG1, JAG2, MAML3, CREBBP, MAML2, CTBP2, DTX2, CTBP1, KAT2A, NUMBL, DLV3, DTX1, and NCSTN) as risk factors using the LASSO regression model (Figures 7(d) and 7(e)). All the KIRC cases were further divided into two groups based on the best cut-off values of the risk scores, i.e., high-risk group and low-risk group. On this basis, we analyzed the differences in survival curves between the two groups and further analyzed the relationship between the grouping model and pathological features of KIRC. Similar to the association between Notch score and KIRC survival, the results show that KIRC survival is significantly better in the low-risk than in the high-risk group (Figure 7(g)). Moreover, the tumor, grade, stage, and metastasis of KIRC are closely related to the new model established by us (Figure 7(f)). Finally, the predictive value of the new KIRC prognostic prediction model in the KIRC was analyzed using ROC analysis. The results show that the AUC of 3-, 5-, 7-, and 10-year survival was 0.732, 0.757, 0.78, and 0.796, respectively (Figures 7(h)–7(k)). An AUC value > 0.7 is considered predictive.

### 3.8. Risk Score Was a Risk Factor in KIRC

**Figure 8:**
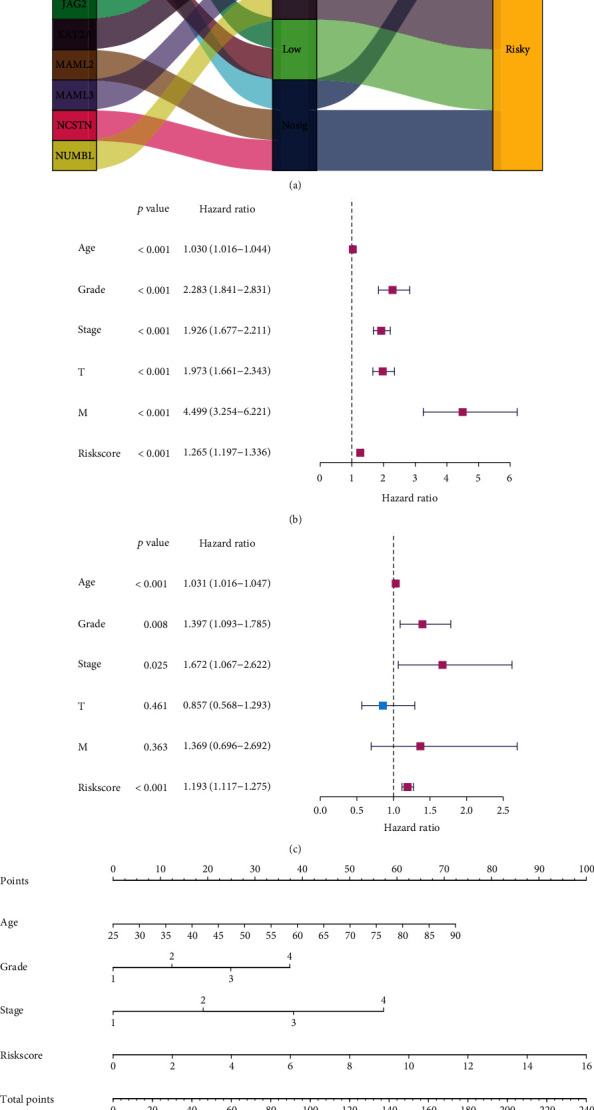
(a) Sankey diagrams plotted for 14 genes. The properties of these genes are represented by Sankey diagrams. (b) Univariate Cox analysis. (c) Multivariate Cox analysis. (d) Nomogram of the model.

According to the previous results of HR analysis, 14 Notch pathway genes as risk factors were divided into risky and protective genes.  Figure 8(a) shows that the high expression groups contains CREBBP, CTBP1, CTBP2, DTX2, JAG1, JAG2, KAT2A, MAML3, and NUMBL; the low expression group included DTX1 and HDAC1; the Nosig group included MAML2, NCSTN, and DVL3. The protective genes include CREBBP, CTBP1, CTBP2, DTX2, JAG1, and DVL3. The remaining genes were all risk genes. The results show that some of the highly expressed genes are protective genes, and some are risk genes, while all low-expression genes are risk genes. The results demonstrate, once again, that the Notch pathway genes may play multiple roles in the tumor. Univariate Cox regression analysis showed that age, grade, stage, T (tumor), M (metastasis), and risk score were risk factors, while multivariate Cox analysis suggested that age, grade, stage, and risk scores were risk factors (Figures 8(b) and 8(c)). Finally, we analyzed the predictive effects of age, grade, stage, and risk score with respect to 5-year survival, 7-year survival, and 10-year survival of KIRC patients using the nomogram of the model (Figure 8(d)). In conclusion, all the results suggest that the Notch risk score is a significant factor for predicting patient prognosis.

### 3.9. Verification by e-MTAB-1980 Dataset in ArrayExpress Database and HPA

**Figure 9:**
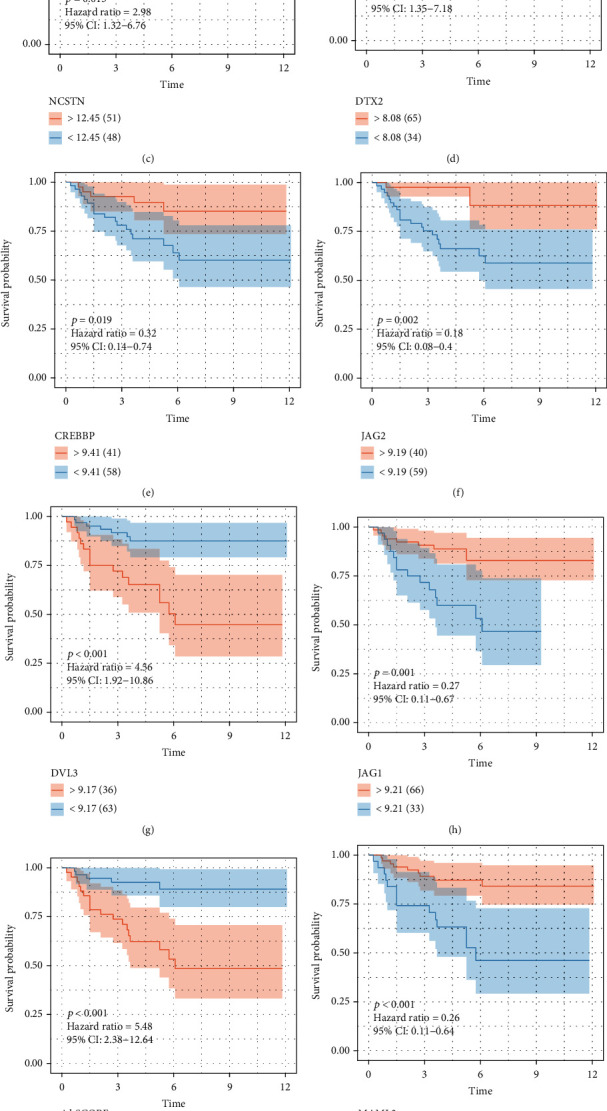
The survival curve of the statistically significant 11 selected Notch pathway proteins in KIRC from e-MTAB-1980 dataset in ArrayExpress database (https://www.ebi.ac.uk/arrayexpress/experiments/E-MTAB-1980/) (^∗^*p* < 0.05, ^∗∗^*p* < 0.01, ^∗∗∗^*p* < 0.001).

**Figure 10:**
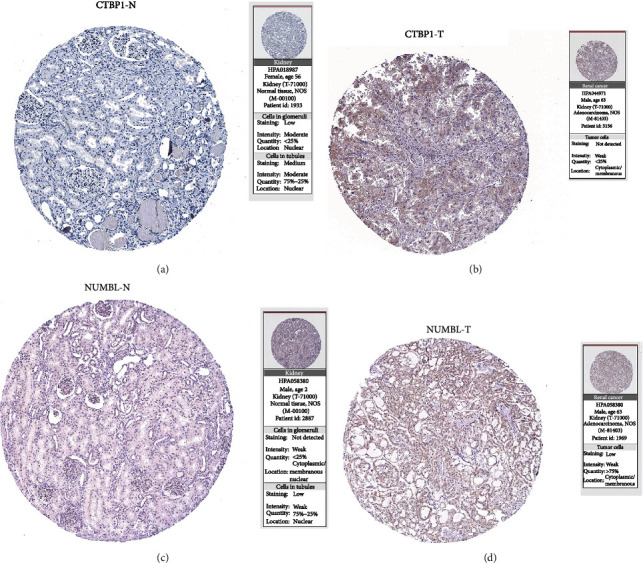
Immunohistochemical images were obtained from online atlas of proteins (https://www.proteinatlas.org/) for CTBP1 and NUMBL, which are representative of the two gene groups (^∗^*p* < 0.05, ^∗∗^*p* < 0.01, ^∗∗∗^*p* < 0.001).

Furthermore, we used the e-MTAB-1980 dataset in the ArrayExpress database for model verification and found model's value with respect to predicting the prognosis of KIRC, which was consistent with TCGA (Figures 9(a)–9(k)). Finally, the relevant online atlas of proteins (https://www.proteinatlas.org/) provided by Sjöstedt et al. [21] and Uhlénet al. [22]was used to verify CTBP1 and NUMBL encoded by selected Notch pathway genes in the model in KIRC. The results show that the expression of CTBP1 and NUMBL in KIRC was significantly higher than that in normal tissue (Figure 10), which suggests that the expression of model proteins is consistent with the expression of the corresponding model genes.

## 4. Discussion

Mammals express four transmembrane Notch receptors (Notch-1, Notch-2, Notch-3, and Notch-4) [35] and five canonical transmembrane ligands (Delta-like1, Delta-like3 Delta-like4, Jagged-1, and Jagged-2) [36–38]. Cell-to-cell contact is generally necessary for the activation of Notch signaling [ 39], generating a short-lived intermediate that is further cleaved by the c-secretase complex [40]. After Notch activation, NIC translocates to the nucleus and binds to CSL (CBF-1, Suppressor of Hairless, LAG1), displacing corepressors, and recruiting coactivators such as MAML proteins [41, 42]. Notch activates numerous genes associated with differentiation and/or survival, including the HES and HEY family [43], cyclin D1 [44], and c-Myc [ 45].

In 2000, Rae et al. compared renal cancer tissues with normal kidney tissue by differential PCR and found that the transcript levels of Notch3 were increased in RCC, which may be involved in the occurrence and progression of tumors [46]. Overexpression of Notch1 increases the risk of distant metastasis in stage T1 RCC [47]. Recently, through the analysis of the Tumor Genome Atlas (TCGA), the low expression of ADAMl7, a key factor involved in Notch signaling enzyme digestion, was found in patients with clear cell carcinoma, chromophile cell carcinoma, or papillary cell carcinoma, suggesting a good prognosis, indirectly suggesting that the Notch pathway may affect the outcomes of patients with multiple types of RCC [48].

Specific inhibition of Notch1 in renal carcinoma cells reduced the level of B-cell lymphoma/leukemia-2 (BC1-2) proteib and increased apoptosis. Meanwhile, the phosphorylation of phosphatidylinositol-3kinase (P13K)/protein kinase B (Akt), which is involved in promoting cell growth and proliferation, decreased [49], suggesting that Notch1 simultaneously regulates the proliferation and apoptosis of tumor cells and inhibits Notch1 signaling, which may be a new therapeutic target. Von Hippe1 is known to cause protein inactivation. The Lindau syndrome (VHL) gene mutation is the most common cause of renal clear cell carcinoma. However, in animal models, VHL deletion alone does not effectively induce renal carcinoma. Moreover, the overexpression of Notch1 intracellular segment in VHL knockout mice revealed accumulation of intracellular fat, cytoplasmic dysplasia nests, and upregulated expression of Hey1 and Hey2 downstream of the Notch pathway, similar to human renal clear cell carcinoma. Therefore, it can prove that the abnormal Notch1 signaling pathway is involved in the pathogenesis of early renal cancer [50].

Angiogenesis and tumor stem cell mechanisms play a role in the pathogenesis of RCC. Multiple studies have shown that the expression of Notch-related ligand DLL4 is elevated in surgical specimens of renal clear cell carcinoma and is an independent prognostic factor. The expression of DLL4 was 9-times higher in the vascular endothelium of RCC tissues (compared to that in the normal renal tissue). DLL4/Notch/Heyl/matrix metalloproteinase (MMP)-9 cascades promote the distant metastasis of the tumor, and lentivirus short hairpin RNA (shRNA) specifically silenced DLL4 in mice, which significantly inhibited the growth of transplanted tumors [51–53]. CDl33 +/CD24 + cells with tumor stem cell properties were isolated from the renal carcinoma cells, ACHN and AKI-L, and treated with a Notch pathway inhibitor (MRK-003). The expression of dry markers, such as copper transporter (CTR2), BC1-2, oct4-binding protein (OCT4), Kruppe1-like factor 4 (KLF4), and multidrug resistance gene (MDRl), was downregulated, and the abilities of self-renewal, tumor formation, invasion, and migration were reduced, and the sensitivity to sorafenib and cisplatin increased [54]. In another study, the inhibition of the Notch signaling pathway by the *γ*-secretase inhibitor, LY3039478, in 769-P and aki-L cells (cell lines originating from highly aggressive RCC), resulted in slowed cell proliferation and downregulated expression of Myc and Cyc1 in Al. Thus, Notch may be a new therapeutic target for advanced renal cancer [55].

In our study, we first investigated the expression of Notch pathway genes in 32 cancers and their differential expression levels. We found that in 32 types of malignant tumors, most tumors showed no CNV gain and loss of Notch genes, and most CNVs fluctuated around 0.2 (Notch genes with CNV gain frequency > 0.04, and Notch genes with CNV loss frequency > 0.05). In addition, we found that most of the Notch pathway genes had SNV in UCEC, and the SNV frequency was almost greater than 0.04. Some of the Notch pathway genes in DLBC SKCM, COAD, STAD, BLCA, CESC, ESCA, HNSC, LUAD, and READ had SNV, and the SNV frequency was greater than 0.04. The SNV frequency of Notch pathway genes in tumors was less than 0.02. We further observed mutations in the Notch pathway genes in KIRC and found that CNV and SNV of the Notch gene in KIRC were significantly low, fluctuating between 0 and 0.02. To determine whether there are compounds that may target or regulate the Notch pathway genes, we identified 20 related compounds that acted on 16 kinds of malignant tumors by compound enrichment score. Furthermore, we used CMap MoA to analyze the mechanism of action of the six compounds and found that each of the six compounds had an independent mechanism of action. We further divided the Notch pathway genes into protective genes and risky genes using information from TCGA and investigated the relationship between Notch pathway gene expression and patient survival. We found that most of the genes of the Notch pathway had no value in tumors, and most of the remaining genes were risk genes, and a few were protective genes. In KIRC, we found an interesting phenomenon in which both protective genes and risk genes were 12, which was also consistent with previous reports. These results indicate that the Notch pathway gene expression is stable in renal clear cell carcinoma without obvious mutations. In addition, the risk genes and protective genes were equally matched in KIRC, indicating that the occurrence of KIRC was related to the proportion between the expression levels of risk genes and protective genes. If the risk genes were dominant, renal cancer would occur, whereas the opposite occurred when the protective genes were dominant.

We further studied the relationship between Notch pathway genes and KIRC, according to the mRNA expression level of the Notch pathway genes obtained from TCGA, and found that there were significant differences in the survival rates of KIRC patients in the three clusters. Cluster 3 was better than cluster 2, while cluster 2 was better than cluster 1. Most Notch genes were highly expressed in KIRC, and patients with high Notch scores survived for longer. These data suggest that Notch may play a role as a tumor suppressor gene in KIRC. Finally, using information from TCGA, we analyzed the relationship between the Notch scores and the clinicopathological features of KIRC. We found that Notch scores were significantly related to T (tumor), stage, metastasis, and fustat of KIRC, and a higher Notch score was associated with lower tumor grade, stage, and prognosis. This further suggests a protective role of the Notch pathway in KIRC. Based on previous reports on the mechanism of Notch in KIRC, and through our analysis of the relationship between Notch pathway genes and the survival of KIRC patients, we found that most Notch pathway genes had protective effects in KIRC.

Presently, targeted drug therapy is the first-line treatment for patients with advanced renal cancer, and its therapeutic effect has been widely recognized. There are also some reports on the effect of targeting the Notch pathway in cancer treatment. Therefore, we performed a GDSC analysis to confirm the effects of some of the most commonly targeted Notch pathway gene drugs in KIRC therapy. Our findings are expected to lead to a better understanding of the correlation between the therapeutic effects of commonly used targeted drugs and Notch genes, which may be helpful for advanced KIRC treatment in the future. Our results show that there is a correlation between IC_50_ and Notch score in some drugs, while there is no significant correlation in the other drugs, which may be related to the difference between the targets of the drugs and the abnormal genes of the Notch pathway in renal cancer. The lower the IC_50_, the more effective a drug will be if it has a target that matches or inhibits the Notch genes, which could cause KIRC.

Currently, there are many studies based on cancer immunotherapy. It is gradually gaining acceptance in the treatment of cancer by intervening in histone acetylation and modulating T cell killing [56, 57]. Furthermore, for KIRC immunotherapy be successful, it is important to clarify the mechanism of action of classical protooncogenes, tumor suppressor genes (KRAS, VHL, etc.), and immune-related genes, especially histone acetylation in KIRC and their relationship with the Notch pathway genes (three clusters). Our results show that HRAS, AKT1, MYC, and VEGFA are highly expressed in cluster 3 but had low expression in cluster 1 in KIRC, suggesting their role as oncogenes. However, common tumor suppressor genes, such as VHL and PTEN, were highly expressed in cluster 3 but had low expression in cluster 1, similar to the performance of the oncogenes. Based on the characteristics of the substrates, it is speculated that human Sirtuin may be involved in regulating the balance between cell survival and death under stress conditions on the one hand, and metabolism regulation, on the other hand, affecting the development, differentiation, aging, and other physiological processes, and is closely related to cancer [58, 59]. In addition, the results of the relationship between Notch pathway genes and Sirtuin family genes show that SIRT1 was highly expressed in cluster 3 but had low expression in cluster 1. SIRT2 and SIRT3 were highly expressed in cluster 2 but with low expression in clusters 1 and 3, with no significant difference. SIRT4, SIRT5, SIRT6, and SIRT7 were all highly expressed in cluster 3 but had low expression in cluster 1. This suggests that the Sirtuin family plays different roles in KIRC. Furthermore, the results of the relationship between the Notch pathway genes and HDAC family genes show that DNMT1, HDAC1-HDAC7, and HDAC9 are all highly expressed in cluster 3 but had low expression in cluster 1. HDAC8 and HDAC11 were highly expressed in cluster 1 but had low expression in cluster 3. HDAC10 had the highest expression in cluster 2 and higher expression in cluster 1 than in 3, like with the Sirtuin family, it is possible that the HDAC family also plays different roles in KIRC. In our study, we further clarified the relationship between the Notch pathway genes and KIRC immune infiltration by analyzing the correlation between the Notch pathway and immune cell infiltration, which may provide a theoretical basis for future immunotherapy of KIRC. Our results show that PTCRA, MFNG, LFNG, Notch1-3, DTX3L, DTX2, CIR1, APH1A, and ADAM17 are positively correlated with most immune-infiltrating agents. In contrast, SNW1, RFNG, NUMB, Notch4, MAML3, KAT2A, DVL2, DVL1, DLL1, and CTBP2 are negatively correlated with immune infiltration. Most immune-infiltrating agents were positively correlated with the Notch pathway genes, including type-II-IFN response, mast cells, DCs, and CCR. A few, including DCs, Tfh, Th2-cells, and T-cell-coinhibition, are negatively correlated with the Notch pathway genes. Finally, we found that DCs, mast cells, T-helper cells, and type-I-IFN responses were all positively correlated with the Notch score.

To further clarify the role of Notch gene-related models in predicting the prognosis of patients with KIRC, the differential expression of 47 Notch pathway genes was studied, and 14 risk genes in the Notch pathway genes were screened out by LASSO regression to construct a model to predict the survival rate of patients with KIRC. First, all KIRC cases were further divided into two groups based on the best cut-off values of the risk scores: high-risk group and low-risk group; second risk group. Secondly, we analyzed the differences in the survival curves between the two groups and further analyzed the relationship between the grouping model and pathological features of KIRC. Similar to the association between Notch score and KIRC survival, the results show that KIRC survival is significantly better in the low-risk group than in the high-risk group. Moreover, the tumor, grade, stage, and metastasis of KIRC are closely related to our new model. Finally, the predictive value of the new KIRC prognostic prediction model in the KIRC was analyzed using ROC analysis. The results show that the AUC was 0.732, 0.757, 0.78, and 0.796, for 3-, 5-, 7-, and 10-year survival, respectively. An AUC value > 0.7 is considered predictive. We hope that this prediction model will be useful in future clinical studies.

To verify the model, the e-MTAB-1980 dataset in the ArrayExpress database was used for model verification, and relevant online atlas of proteins verified whether the expressions of model's proteins were consistent with the expressions of the corresponding model's genes. The results demonstrate that the value of the model with respect to predicting the prognosis of KIRC was consistent with TCGA. Meanwhile, our results show that the expression of CTBP1 and NUMBL in KIRC was significantly higher than that in normal tissue, suggesting that the expression of model proteins is consistent with the expression of the corresponding model's genes. As explained above, the Notch-related prognosis model of ccRCC using LASSO regression is an effective way to predict the prognosis of KIRC, providing a relatively meaningful strategy for the treatment of KIRC.

In conclusion, the pathogenesis of KIRC is associated with an abnormally activated Notch signaling pathway, and its inhibition of this pathway may be a potential drug target. Currently, studies in this field mainly focus on Notch1 and its DLL receptors, which are closely related to angiogenesis. However, there are few studies on the role of Notch ligands and receptor proteins in promoting angiogenesis, abnormal lipid metabolism, invasion, and metastasis in renal cancer, which may become the direction of basic research in the future.

## Data Availability

The data used to support the findings of this study are available from the corresponding authors upon request.

## Abbreviations

KIRC: Kidney renal clear cell carcinoma
RCC: Renal cell carcinoma
THCA: Thyroid carcinoma
THYM: Thymoma
PRAD: Prostate adenocarcinoma
UCEC: Uterine corpus endometrial carcinoma
SKCM: Skin cutaneous melanoma
COAD: Colon adenocarcinoma
TCGA: The Cancer Genome Atlas
GDSC: Genomics of Drug Sensitivity in Cancer
HPA: The Human Protein Atlas
LASSO: Least absolute shrinkage and selection operator
GSEA: Gene set enrichment analysis
CMap: Connectivity map
CNV: Copy number variation
SNV: Single-nucleotide variation
WNT-*β*-catenin: Wingless/integrated-*β*-catenin
TP53: Tumor protein 53
VEGF: Vascular endothelial growth factor
TME: The tumor microenvironment
HMGCR: Recombinant 3-hydroxy-3-methylglutaryl coenzyme a reductase
HRAS: HRas protooncogene
MYC: Myelocytomatosis oncogene
VEGFA: Vascular endothelial growth factor-A
VHL: Von Hippel-Lindau
PTEN: Phosphatase and tensin homolog
BRAF: Mutant serine/threonine protein kinase B-Raf
KRASM: KRAS-mutant
TOR: Target of rapamycin
EGFR: Epidermal growth factor receptor
CCND1: CyclinD1
CTNNB1: Catenin beta1
STAT3: Signal transducer and activator of transcription 3
HDAC: Zinc-dependent histone deacetylase
SIRT: Sirtuins
SDHA: Succinate dehydrogenase subunit A.
